# Diagnostic and therapeutic strategies for non-alcoholic fatty liver disease

**DOI:** 10.3389/fphar.2022.973366

**Published:** 2022-11-02

**Authors:** Yajie Fu, Yanzhi Zhou, Linhu Shen, Xuewen Li, Haorui Zhang, Yeqi Cui, Ke Zhang, Weiguo Li, Wei-dong Chen, Shizhen Zhao, Yunfu Li, Wenling Ye

**Affiliations:** ^1^ Key Laboratory of Receptors-Mediated Gene Regulation, Hebi Key Laboratory of Liver Disease, School of Basic Medical Sciences, The People’s Hospital of Hebi, Henan University, Kaifeng, China; ^2^ Key Laboratory of Receptors-Mediated Gene Regulation and Drug Discovery, School of Basic Medical Science, Inner Mongolia Medical University, Hohhot, China

**Keywords:** NAFLD, NASH, diagnostic, therapeutic strategies, drug development

## Abstract

The global incidence rate of non-alcoholic fatty liver disease (NAFLD) is approximately 25%. With the global increase in obesity and its associated metabolic syndromes, NAFLD has become an important cause of chronic liver disease in many countries. Despite recent advances in pathogenesis, diagnosis, and therapeutics, there are still challenges in its treatment. In this review, we briefly describe diagnostic methods, therapeutic targets, and drugs related to NAFLD. In particular, we focus on evaluating carbohydrate and lipid metabolism, lipotoxicity, cell death, inflammation, and fibrosis as potential therapeutic targets for NAFLD. We also summarized the clinical research progress in terms of drug development and combination therapy, thereby providing references for NAFLD drug development.

## 1 Introduction

Non-alcoholic fatty liver disease (NAFLD) is a liver disease, which encompasses diffuse non-alcoholic liver steatosis, non-alcoholic steatohepatitis (NASH), and other features of liver damage, such as liver cirrhosis and hepatocellular carcinoma (Friedman et al., 2018a). NAFLD is defined by the presence of steatosis in more than 5% of liver cells and in the absence of excessive alcohol consumption (men ≥ 30 g per day and women ≥ 20 g per day) or other chronic liver diseases (Cobbina and Akhlaghi, 2017). In addition, NASH can occur if steatosis is accompanied by inflammation and hepatocyte ballooning (Yeh and Brunt, 2014). NAFLD has become increasingly common in recent years, and it is now the leading cause of chronic liver disease in many countries. The pathogenesis of NAFLD has not yet been fully elucidated; however, obesity, type 2 diabetes mellitus (T2DM), and other metabolic disorders (Akhtar et al., 2019) play key roles in increasing the incidence and prevalence of NAFLD. The global prevalence of NAFLD is approximately 25.2%, and the overall prevalence of NASH is approximately 1.5%–6.5%. People with obesity are more likely to develop NAFLD. Approximately 50% and 80% of patients with NAFLD and NASH, respectively, are overweight. Patients with T2DM also have a high prevalence of ΝAFLD (56%–59%), and the prevalence of NASH in patients with T2DM is 37% (Younossi et al., 2019a). The prevalence of NAFLD and NASH in patients with dyslipidemia has also increased significantly (Wu et al., 2016). NAFLD is a multisystem disease; however, the only clinical symptom is liver disease. It also affects the cardiovascular, excretory, and endocrine systems and carries the risk of extrahepatic malignant tumors. The most common cause of death in NAFLD is cardiovascular disease, followed by malignant tumors and liver-related complications (Li and Ahmed, 2020).

The pathogenesis of NAFLD is un-clear, and the “two-hit” theory is widely accepted in the early stage. This theory suggests that lipid metabolism disorder triggers the “first blow” and causes fatty degeneration of liver parenchymal cells. On this basis, a “second blow” can occur, wherein oxidative stress and mitochondrial dysfunction take place in the liver with over-deposition of lipids, forming lipid peroxide products and eventually damaging liver cells (Day and James, 1998). Further research found, that the two-hit theory cannot fully explain the pathogenesis of NAFLD, and therefore, the “multiple-hit” theory has gradually attracted attention. At the same time, available evidence suggests that genetic factors are also important in the pathogenesis of NAFLD. Family studies show that the risk of NAFLD is significantly higher in patients who have first-degree relatives affected by this disease compared with the normal population. (Del Campo et al., 2018). Over 100 loci have been examined in genome-wide association studies and candidate gene studies, and five genes (PNPLA3, TM6SF2, GCKR, MBOAT7, and HSD17B13) have been identified as reliably and robustly predisposing individuals to MAFLD. (Valenti and Baselli, 2018; Sookoian and Pirola, 2019). All five genes are involved in glucose and fat homeostasis regulatory pathways. Although the current study of these mechanisms is incomplete, these findings may provide some scientific information for targeted therapy of NAFLD (Carlsson et al., 2020). Due to its complex pathogenesis and absence of specific drugs, NAFLD treatment mainly involves lifestyle intervention. However, the efficacy of this treatment is limited. This article summarizes the diagnostic methods and therapeutic drugs in development for NAFLD, including the targets that regulate each pathway. This review also outlines the current state of NAFLD drug development and combination therapy.

## 2 Diagnostic progress

The current diagnostic methods for NAFLD include ultrasound diagnosis, computed tomography (CT), magnetic resonance imaging (MRI), liver biopsy, and laboratory testing. When NAFLD is suspected, the patient should first be evaluated using non-invasive imaging. Although ultrasound has a high detection rate for moderate and severe liver steatosis, its sensitivity and specificity are low for mild liver steatosis. Hence, ultrasound cannot be used to diagnose steatohepatitis or fibrosis (Bril et al., 2015). CT can also be used to diagnose NAFLD with the same level of sensitivity and specificity as ultrasound. Hyodo et al. (Hyodo et al., 2017) found that the multi-substance analysis algorithm of dual-energy CT allows for quantitative evaluation of liver steatosis. However, this is expensive and involves exposure to radiation, which limits its clinical application. MRI is the most sensitive detection method and can detect approximately 5% of hepatocyte steatosis. However, its accuracy may be affected by fibrosis and severe steatosis (Lee and Kim, 2019). Liver biopsy is the “gold standard” for NAFLD diagnosis, and can be used to accurately assess liver cell inflammation, liver fibrosis, and liver steatosis (Nalbantoglu and Brunt, 2014). When non-invasive imaging is not sufficient to accurately diagnose NAFLD, liver biopsy can assist in the diagnosis. Meanwhile, laboratory testing can predict NAFLD early and assess the risk and prognosis of cardiovascular and other dangerous events in patients with NAFLD; therefore, it is also worthy of attention.

The most important histological feature of advanced NAFLD is fibrosis (Chalasani and Younossi, 2018). Therefore, the evaluation of liver fibrosis is essential. The imageological examinations mentioned above do not reliably reflect the progression of liver fibrosis in patients with NAFLD. Consequently, there has been significant interest in developing noninvasive biomarkers and clinical prediction rules. Simple biochemical markers of fibrosis such as low albumin, prolonged prothrombin time and thrombocytopenia are markers of advanced cirrhosis. These biochemical markers are non-invasive and inexpensive, but they are less reliable (Jennison et al., 2019). Currently, several methods have been developed to assess hepatic fibrosis, such as the NAFLD Fibrosis Score (NFS), Fibrosis-4 (FIB-4) Score and Enhanced Liver Fibrosis (ELF) test (Shah et al., 2009). The NFS is based on six variables: age, BMI, hyperglycemia, platelet count, albumin, and AST/ALT ratio. FIB-4 index is an algorithm based on platelet count, age, AST, and ALT (Kaswala et al., 2016). A previous study showed that NFS and FBI-4 were better than other indices such as AST to Platelet Ratio Index (APRI) and AST/ALT ratio in predicting advanced fibrosis (Imajo et al., 2016). The Enhanced Liver Fibrosis (ELF) panel consisting of plasma levels of three matrix turnover proteins (tissue inhibitor of metalloproteinase 1, hyaluronic acid and N-terminal procollagen III-peptide) have an AUROC of 0.90 with 90% specificity and 80% sensitivity for detecting advanced fibrosis (Angulo et al., 2007). In addition, ELF is approved for commercial use in Europe.

## 3 Therapeutic targets and related drugs for NAFLD

**FIGURE 1:**
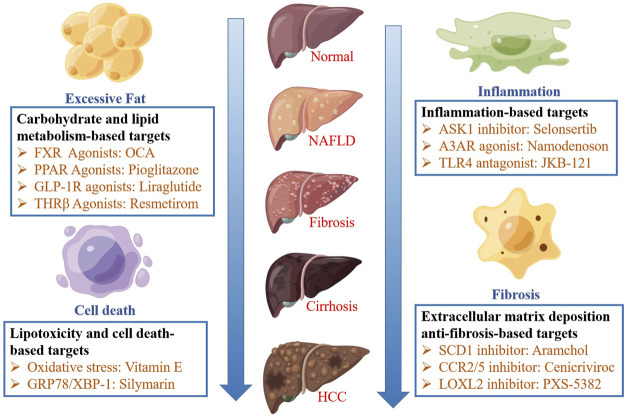
The pathophysiology and therapeutic strategies of NAFLD. The therapeutic targets of NAFLD can be divided into four categories: carbohydrate and lipid metabolism-based, lipid toxicity and cell death-based, inflammation-based, and extracellular matrix deposition anti-fibrosis-based targets.

Studies show that the therapeutic targets of NAFLD can be divided into four categories: carbohydrate and lipid metabolism-based, lipid toxicity and cell death-based, inflammation-based, and extracellular matrix deposition anti-fibrosis-based targets. Various drugs can improve NASH by acting on different targets. These therapeutic targets and their related drugs are schematically depicted in Figure 1.

### 3.1 Carbohydrate and lipid metabolism-based targets

Excessive fatty acids in the liver lead to excess energy and the production of lipotoxic metabolites by hepatocytes, thereby damaging hepatocytes (Neuschwander-Tetri, 2010). Therefore, reducing intrahepatic free fatty acids is a potential strategy for NAFLD treatment, which can be achieved by increasing insulin sensitivity, fatty acid oxidation, or fatty acid export and storage by peripheral tissues and reducing *de novo* lipogenesis (DNL). The research progress on drugs targeting carbohydrate and lipid metabolism is summarized in Table 1.

**TABLE 1:** Mechanism and Research Progress of drugs targeting carbohydrate and lipid metabolism.

Drug name	Drug targets	NCT number	Current research progress	Status	Reference
Dapagliflozin	SGLT2 Inhibitors. Improve steatosis and fibrosis	NCT05459701	Phase Ⅳ	Recruiting	https://clinicaltrials.gov
Liraglutide	GLP-1 Receptor Agonist. Increase fatty acid oxidation and reduce *de novo* lipogenesis	NCT02654665	Phase Ⅲ	Completed	Khoo et al. (2019)
Oltipraz	LXR Agonist. Inhibit fatty acid synthesis through AMPK-S6K1 pathway and LXR-SREBP-1c pathway in liver	NCT02068339	Phase Ⅲ	Completed	https://clinicaltrials.gov
Resmetirom (MGL-3196)	Selective Thyroid Hormone Receptor *ß* Agonist. Reduce liver fat and lipoprotein	NCT04951219, NCT03900429	Phase Ⅲ	Recruiting	https://clinicaltrials.gov
Elafibranor	PPAR-α/δ Agonist. Improve insulin sensitivity, glucose homeostasis, and lipid metabolism and reduce inflammation	NCT02704403	Phase Ⅲ	Terminated	https://clinicaltrials.gov
6-ethylchenodeoxycholic acid (OCA)	FXR Agonist. Induce the expression of intestinal sex hormones, especially fibroblast growth factor 19. Improve liver blood index and reduce liver fibrosis	NCT02548351	Phase Ⅲ	Not yet recruiting	https://clinicaltrials.gov
Semaglutide	GLP-1 Receptor Agonist. Increase fatty acid oxidation and reduce *de novo* lipogenesis	NCT02970942,NCT05067621	Phase Ⅱ/Ⅲ	Completed/Not yet recruiting	Newsome et al. (2021)
Omega-3 polyunsaturated fatty acids	Inhibit hepatic *de novo* lipogenesis and increase fat oxidation	NCT00681408	Phase Ⅱ/Ⅲ	Completed	Argo et al. (2015)
Synbiotic	Improve insulin resistance by changing gut microbiota	NCT01791959	Phase Ⅱ/Ⅲ	Completed	Eslamparast et al. (2014)
Licogliflozin	SGLT2 Inhibitors. Improve steatosis and fibrosis	NCT03205150	Phase Ⅱ	Completed	https://clinicaltrials.gov
Vitamin D	Regulate oxidation, improve inflammation, lipotoxicity, and fibrosis	NCT01571063,NCT01792115	Phase Ⅱ	Completed	https://clinicaltrials.gov
Px-104	FXR Agonist. Improve insulin sensitivity and liver enzymes	NCT01999101	Phase Ⅱ	Completed	Traussnigg et al. (2021)
Cilofexor	FXR Agonist. Reduce hepatic steatosis and serum bile acid	NCT02854605	Phase Ⅱ	Completed	Patel et al. (2020)
EDP-305	FXR Agonist. Reduce inflammation and liver fat content	NCT03421431	Phase Ⅱ	Completed	Ratziu et al. (2022)
Ervogastat (PF-06865571)	DGAT Inhibitors. Reduced liver fat fraction	NCT03776175	Phase Ⅱ	Completed	https://clinicaltrials.gov
Saroglitazar	PPAR-α/γ Agonist. Improve insulin resistance and increase lipid oxidation	NCT03061721, NCT03863574	Phase Ⅱ	Completed	https://clinicaltrials.gov
VK-2809 (MB07811)	Selective Thyroid Hormone Receptor *ß* Agonist. Reduce liver fat and lipoprotein	NCT04173065	Phase Ⅱ	Recruiting	https://clinicaltrials.gov
TERN-501	Selective Thyroid Hormone Receptor *ß* Agonist. Reduce liver fat and lipoprotein	NCT05415722	Phase Ⅱ	Recruiting	https://clinicaltrials.gov
ION224 (IONIS-DGAT2_Rx_)	DGAT Inhibitors. Reduced liver fat fraction	NCT04932512	Phase Ⅱ	Recruiting	https://clinicaltrials.gov
ASC40(TVB-2640)	FASN Inhibitor. Reduce excessive fat in the liver and inhibit inflammation and fibrosis	NCT04906421	Phase Ⅱ	Recruiting	https://clinicaltrials.gov
Tropifexor (LJN452)	FXR Agonist. Reduced steatohepatitis, fibrosis, and profibrogenic gene expression	NCT02855164	Phase Ⅱ	Terminated	https://clinicaltrials.gov
Nidufexor (LMB763)	FXR Agonist. Reduce steatosis, inflammation and fibrosis	NCT02913105	Phase Ⅱ	Terminated	https://clinicaltrials.gov
Seladelpar	PPAR-δ Agonist. Improve glucose homeostasis, lipid metabolism and reduce inflammation	NCT03551522	Phase Ⅱ	Terminated	https://clinicaltrials.gov
MET-409	FXR Agonist. Reduce hepatic steatosis, inflammation and fibrosis	NCT04702490	Phase Ⅱ	Not yet recruiting	https://clinicaltrials.gov
PXL065	PPAR-γ Agonist. Increase the levels of plasma adiponectin, thus playing anti-inflammatory and anti-fibrosis roles	NCT04321343	Phase Ⅱ	Not yet recruiting	https://clinicaltrials.gov
ASC41	Selective Thyroid Hormone Receptor *ß* Agonist. Reduce liver fat and lipoprotein	NCT05462353, NCT05118360	Phase Ⅱ	Not yet recruiting	https://clinicaltrials.gov
BAR502	Steroidal dual ligand for FXR and GPBAR1.Promote adipose tissue browning, prevent liver injury caused by HFD.	NCT05203367	Phase Ⅰ	Not yet recruiting	https://clinicaltrials.gov
Dulaglutide	GLP-1 Receptor Agonist. Increase fatty acid oxidation and reduce *de novo* lipogenesis	NCT03590626	Clinical phase	Completed	Kuchay et al. (2020)
InT-767	FXR Agonist. Restore lipid and glucose metabolism, reduce insulin resistance and inhibit TNF- *a* And NF- κ B to attenuate the pro-inflammatory response	-	Preclinical study	Completed	Hu et al. (2018b)
Altenusin	FXR Agonist. Reduce body weight and fat mass, reduce blood glucose, and reverse HFD induced liver lipid droplet accumulation and bullous steatosis	-	Preclinical study	Completed	Zheng et al. (2017)
Yangonin	FXR Agonist. Activate FXR signal to inhibit hepatic lipogenesis and gluconeogenesis, promote lipid metabolism and glycogen synthesis, and improve insulin sensitivity	-	Preclinical study	Completed	Dong et al. (2019)
Isotschimgine	Improved steatosis and inflammation and fibrosis	-	Preclinical study	Completed	Li et al. (2021)

PPAR, peroxisome proliferator-activated receptor; GLP-1RA, glucagon-like peptide receptor agonist; SGLT, sodium-glucose cotransporter; FXR, farnesoid X receptor; TNF-α, α-tumor necrosis factor; NF-κB, Nuclear factor kappa-B; HFD, High-fat diet; HSC, hepatic stellate cell; TG, triglyceride; MCD, methionine and choline deficient.

#### 3.1.1 Farnesoid X receptor agonists

The farnesoid X receptor (FXR) is a bile acid receptor that regulates bile acid absorption, metabolism, and secretion and is closely associated with the development of cholestasis, fatty liver disease, cholesterol stones, enteritis and tumors (Koutsounas et al., 2015). FXR shares a common architecture with classical nuclear receptors, which are composed of an N-terminal ligand-independent activation domain, conserved DNA-binding domain (DBD), and C-terminal ligand-binding domain (LBD) (Weikum and Liu, 2018). FXR agonists can improve insulin sensitivity, inhibit DNL and reduce bile acid synthesis (Neuschwander-Tetri et al., 2015; Harrison et al., 2018b). In addition, FXR activation can inhibit sterol regulatory element-binding protein 1c (SREBP-1c), a key transcription factor for lipid regeneration that regulates triglyceride metabolism and lipid regeneration (Watanabe et al., 2004). Therefore, FXR is considered the most promising target for NAFLD treatment.

##### 3.1.1.1 Obeticholic acid

Obeticholic acid (OCA) is a steroidal FXR agonist that has completed phase Ⅲ clinical trials for NASH treatment. As its safety and effectiveness are still not guaranteed, the FDA rejected a new application for NASH treatment submitted by Intercept Pharmaceuticals Inc. in 2019 (Younossi et al., 2021). Pruritus, a common symptom of cholestatic diseases, and is a common side effect of FXR agonists (Srivastava, 2014; Hirschfield et al., 2015; Kowdley et al., 2018). Generally, mild pruritus occurs at the beginning of OCA treatment and does not deteriorate over time (Younossi et al., 2019b).

Recent studies show that low doses of OCA are a safe and effective treatment for NASH and cholestatic liver disease. A double-blind study showed that OCA can improve NASH fibrosis [OR: 1.95 (1.47–2.59; *p* < 0.001)]. The probability of improvement was 1.61 (1.03–2.51; *p* = 0.03) in the 10 mg OCA dose group and 2.23 (1.55–3.18; *p* < 0.001) in the 25 mg OCA dose group. However, in patients with NASH, 25 mg OCA resulted in significant adverse events and drug discontinuation reactions compared with 10 mg OCA [0.95 (0.6–1.5); *p* = 0.84] [2.8 (1.42–3.02); *p* < 0.001]. OCA (5 mg) was associated with the lowest risk of itching (Kulkarni et al., 2021). Another study showed that compared with the placebo, OCA increased the liver transport of bound bile acid tracer 11C CSAR, thereby increasing the transport of endogenous bound bile acids from hepatocytes to bile ducts. Therefore, OCA shortens the exposure time of hepatocytes to potentially cytotoxic bile acids and reduces hepatocellular injury (Kjærgaard et al., 2021). In conclusion, as a representative steroid FXR agonist, OCA should be considered in terms of safety and selectivity.

##### 3.1.1.2 Tropifexor

Tropifexor, also known as LJN-452, is a highly potent non-steroidal FXR agonist. It can activate FXR and regulate FXR target genes at very low doses and upon systemic exposure. Studies show that the efficacy of tropifexor at a dose of <1 mg/kg is superior to that 25 mg/kg OCA in the liver of insulin-resistant NASH obesity models (islet amyloid liver NASH model and chemical and dietary models of NASH stelic animal model) (Hernandez et al., 2019). Some steroidal FXR agonists are agonists of GPBAR1 (also known as TGR5), another bile acid receptor that is the main cause of side effects, including pruritus (Kowdley et al., 2018). Unlike steroidal drugs, non-steroidal drugs do not usually interact with non-target proteins, such as GPBAR1; that is, they have higher selectivity. A previous study showed that the EC_50_ of OCA on GPBAR1 was 0.918 μM, whereas tropifexor had no detectable activity on GPBAR1 (EC_50_ > 10 μM) (Tully et al., 2017). Moreover, in clinical trials, itching was not observed in healthy volunteers within 2 weeks of daily administration (Badman et al., 2020). Phase Ⅱ clinical trials for the evaluation of the safety and efficacy of tropifexor in treating primary biliary cirrhosis and NASH are currently ongoing (Kowdley et al., 2020). The crystal structure of the FXR-tropifexor complex has been resolved, and the molecular mechanism of the combination of tropifexor and FXR-LBD has been proposed (Jiang et al., 2021). In addition, the highly selective structures of tropifexor to FXR and GPBAR1 were analyzed, which provided a better understanding of the development of new compounds targeting FXR (Jiang et al., 2021). As a representative non-steroidal drug, tropifexor is preferable to steroidal drugs in terms of selectivity and is expected to become the first-line drug for NASH treatment.

#### 3.1.2 Peroxisome proliferator-activated receptor agonists

Peroxisome proliferator-activated receptors (PPARs) are ligand-activated receptors in the nuclear hormone receptor family and include three subtypes: PPARα, PPARβ/δ and PPARγ. PPARα is highly expressed in the liver, skeletal muscle, kidney, heart and vascular wall, but at relatively low levels in fatty tissues and cartilage. It regulates fatty acid metabolism, including absorption, transport and *α*-oxidation. PPARβ is widely expressed throughout the body and regulates the *ß*-oxidation of free fatty acids, improving glucose homeostasis and exerting anti-inflammatory effects. PPARγ is expressed in adipose, vascular smooth muscle and myocardial tissues in mammals. It can regulate adipogenesis and fatty acid storage, and improve insulin sensitivity. Therefore, PPARs agonists may be efficacious in NAFLD treatment. Among them, PPAR-α agonists are expected to reduce the metabolic overload of NASH. However, clinical trials of fibrates are not satisfactory (Laurin et al., 1996; Basaranoglu et al., 1999; Fernández-Miranda et al., 2008). Therefore, people begin to study selective PPAR agonists, such as pioglitazone (PPAR- γ agonist), saroglitazar (PPAR- α/γ agonist) (Kumar et al., 2020), elafibranor (PPAR-α/δ agonist) (Ratziu et al., 2016).

##### 3.1.2.1 Pioglitazone

Pioglitazone is a PPARγ agonist that can increase levels of plasma adiponectin, through anti-inflammatory and anti-fibrosis action (Bajaj et al., 2004). To investigate the efficacy and safety of long-term pioglitazone treatment for patients with NASH and T2DM, 101 patients with prediabetes or T2DM were randomly divided into 45 mg/d pioglitazone treatment and placebo groups for 18 months in a randomized, double-blind, placebo-controlled trial (NCT00994682). The results showed that 58%patients treated with pioglitazone had a reduction of at least two points on the NAFLD activity score without worsening fibrosis, and 51% reached a resolution of NASH compared with the placebo group. In addition, the group treated with pioglitazone showed improvement in their fibrosis score, hepatic triglyceride content, and insulin sensitivity (Cusi et al., 2016). However, pioglitazone has some side effects, such as the increased risk of body weight gain, fluid retention, bladder cancer, bone fracture, and increased incidence of hospitalization for heart failure. These side effects may be mitigated by altering the pioglitazone dose (Giles et al., 2008; Satirapoj et al., 2018; Tang et al., 2018; Portillo-Sanchez and Bril, 2019). In summary, on the premise of biopsy-proven NASH, pioglitazone can be used to treat NASH patients with or without T2DM according to guidelines (Chalasani and Younossi, 2018).

##### 3.1.2.2 PXL065

PXL065 is a newly patented deuterium-stabilized R-stereoisomer of pioglitazone. It has anti-inflammatory and anti-NASH properties related to pioglitazone and causes little or no weight gain or fluid retention, thus, it may have a better therapeutic effect on NASH than pioglitazone. To evaluate the efficacy and safety of PXL065 in patients with NASH confirmed by non-cirrhotic biopsies, a 36-week, randomized, double-blind, placebo-controlled phase II clinical trial is in progress (NCT04321343). In this trial, 123 participants were randomly divided into three PXL065 treatment groups (7.5, 15, and 22.5 mg) and a placebo group. The primary endpoint of the trial was the relative change in the percentage of liver fat content measured using MRI-PDFF at 36 weeks. The phase II clinical trial of the PXL065 were completed in August 2022, but no results have been posted on ClinicalTrials.gov at present.

#### 3.1.3 Glucagon-like peptide receptor agonists

Glucagon-like peptide-1 (GLP-1) is an incretin mainly produced by intestinal L cells. Glucagon-like peptide-1 receptor (GLP-1R) agonists can enhance insulin secretion, inhibit glucagon secretion in a glucose concentration-dependent manner, and delay gastric emptying. It can also reduce food intake through central appetite inhibition by activating the GLP-1 receptor, thereby reducing blood glucose levels (Gupta et al., 2010). GLP-1R agonists are anti-diabetic drugs that potentially affect on NAFLD. However, owing to a lack of sufficient evidence, it is premature to consider them for the specific treatment of liver disease in patients with NAFLD or NASH, according to the guidelines of the American Association for the Study of Liver Diseases in 2018 (Chalasani and Younossi, 2018).

##### 3.1.3.1 Liraglutide

Liraglutide, the most widely used GLP-1R agonist, was approved T2DM treatment in 2010 (Drucker et al., 2010). It has been shown to have a potential benefit in preventing cardiovascular events (Marso et al., 2016b). Several clinical trials have reported the potential efficacy of liralutide in patients with NAFLD. A phase II trial (NCT01237119) evaluated the safety and efficacy of liraglutide in the treatment of NASH. 52 patients with NASH were randomly assigned to the liraglutide treatment group (1.8 mg daily) and the placebo group. At week 48, there were fewer patients with fibrosis progression and a higher proportion of patients with improved steatosis and hepatocyte ballooning in the treatment group than in the placebo group. Liraglutide was generally well tolerated by subjects, and the most common side effects were mild, transient gastrointestinal adverse events, such as constipation, diarrhea, and loss of appetite (Armstrong et al., 2016). A phase III trial (NCT02654665) comparing the effects of liraglutide and lifestyle changes on weight loss in patients with NAFLD showed that once-daily liraglutide was as effective as diet and exercise for over 26 weeks in adult patients with NAFLD and obesity. Liraglutide could reduce weight and improve liver steatosis, insulin resistance, and hepatocyte injury; however, these improvements were not sustained after treatment withdrawal (Khoo et al., 2019). Few trials have evaluated the efficacy of liraglutide in patients with NAFLD, and larger trials are needed to evaluate the potential of liraglutide in NAFLD treatment.

##### 3.1.3.2 Semaglutide

Semaglutide, a novel GLP-1R agonist, reduces cardiovascular risk (Marso et al., 2016a) and levels of alanine aminotransferase (ALT) and inflammatory markers (Newsome et al., 2019); it is a potential treatment for NAFLD. A 72-week, double-blind, phase II trial (NCT02970942) was conducted to evaluate the efficacy and safety of semaglutide in patients with NASH. The 320 patients were randomly divided into three treatment groups and three placebo groups. The patients in the treatment groups were subcutaneously injected with 0.1, 0.2, or 0.4 mg semaglutide once daily. The results showed that the proportion of patients with NASH remission treated with semaglutide was significantly higher than those treated with the placebo (Newsome et al., 2021). This study lays a good foundation for larger trials to evaluate the efficacy of Semaglutide on NAFLD in the future.

#### 3.1.4 Omega-3 polyunsaturated fatty acids

The n-3 polyunsaturated fatty acid (n-3 PUFA) family contains several long-chain fatty acids including docosahexaenoic acid (DHA), eicosapentaenoic acid (EPA), stearic acid (SDA), docosahexaenoic acid (DPA), and *α*-linolenic acid (ALA). Humans and other mammals can synthesize EPA and DHA from ALA through a series of desaturases and carbon chain-lengthening enzymes. However, the conversion efficiency is low, and ALA cannot be synthesized in the body and must be absorbed from food. Marine fish are known sources of EPA and DHA (Siriwardhana et al., 2012).

N-3 PUFAs are a potential treatment for NAFLD as they can inhibit hepatic DNL and increase fat oxidation. However, n-3 PUFAs supplementation also leads to a significant increase in fasting and postprandial blood glucose concentration (Green et al., 2020). Several trials have been conducted to study its safety and efficacy in NAFLD treatment (Li et al., 2015; Eriksson et al., 2018; Oscarsson et al., 2018; Manousopoulou et al., 2019; Parker et al., 2019; Cansanção et al., 2020; Climax et al., 2020). In a randomized, double-blind, placebo-controlled trial, 78 patients with NASH were randomly divided into a control group and a PUFA therapy group (50 ml PUFA with a 1:1 ratio of dietary DHA and EHA daily). The results showed that steatosis grade, fibrosis stage, ballooning score, and necrotizing inflammatory grade were significantly improved in the treatment group after 6 months of treatment compared to that in the control group (Li et al., 2015). Moreover, in another randomized, double-blind, placebo-controlled clinical trial, alkaline phosphatase (ALP) and liver fibrosis decreased significantly after 6 months of supplementation with fish oil containing DHA and EPA in patients with NAFLD (Cansanção et al., 2020). Thus, n-3 PUFAs supplementation may improve NAFLD in several ways.

However, in a double-blind randomized controlled trial in 2019, 50 apparently healthy overweight men (BMI 25.0–29.9 kg/m^2^; waist circumference >94 cm) between 18 and 60 years old were randomly divided into a control group receiving olive oil capsules and a PUFA therapy group receiving 1728 mg fish oil per day (containing 588 mg EPA and 412 mg DHA) for 12 weeks. The results showed that compared to the control, PUFA treatment had no significant effect on liver fat or enzymes (Parker et al., 2019). This suggests that n-3 PUFAs do not effectively reduce liver fat in overweight men. Moreover, 1,000 mg of EPA and DHA per day may not be sufficient to reduce liver or visceral fat in apparently healthy men who are overweight and have an increased risk of NAFLD.

Hence, the efficacy of n-3 PUFAs in NAFLD treatment may be heterogeneous in terms of sex and age. Overall, the effects of different treatment dosages, durations, age, sex, and race of the subjects on the efficacy of n-3 PUFAs in the treatment of NAFLD need to be further studied.

#### 3.1.5 Synbiotics

Gut microbiota disorder is a pathogenic factor of NAFLD because it is linked to NAFLD through microbial metabolites and the gut-liver axis. NAFLD progression is regulated by the effect of gut microbiota on intestinal epithelial barrier function, the Toll-like receptor (TLR) signaling pathway, and choline metabolism (Dong and Jacobs, 2019). Therefore, gut microbiota can be a therapeutic target for NAFLD (Zhou and Fan, 2019). At present, gut microbiota therapy is in development; large-scale and well-organized RCT trials are needed to confirm the clinical efficacy of gut microbiota therapy for NAFLD (Ma et al., 2017). Some drugs, including antibiotics, probiotics, prebiotics, and synbiotics, can potentially regulate gut microbiota and thus have a therapeutic effect on NAFLD.

Synbiotics are biological agents that consist of probiotics and prebiotics. Synbiotics may improve insulin resistance by altering the gut microbiota, and trials investigating the efficacy of synbiotics in NAFLD treatment have been conducted (Eslamparast et al., 2014; Mofidi et al., 2017; Bakhshimoghaddam et al., 2018; Scorletti et al., 2020). In a 24-week open-label, randomized controlled clinical trial (IRCT2017020932417N2), 102 overweight [BMI 31.2 ± 4.9 kg/m^2^ (mean ± SD)] patients, including 50 males and 52 females, with an average age of 40, were randomly divided into a control group (no supplementation) and two intervention groups. The patients were administered 300 g synbiotic yogurt containing 10^8^ colony-forming units of *Bifidobacterium* per mL and 1.5 g inulin or conventional yogurt daily. The results show that compared with the conventional and control groups, patients with NAFLD in the synbiotic group had significantly improved liver steatosis and liver enzyme concentration (Bakhshimoghaddam et al., 2018). Moreover, in another randomized, double-blind, placebo-controlled clinical trial (NCT02530138), 50 lean patients with NAFLD (BMI ≤ 25) were randomly divided into a synbiotic group, which was administered synbiotic capsules containing 200 million bacteria of seven strains and fructo-oligosaccharide, and a placebo group which was administered maltodextrin capsules for 28 weeks. The results showed that hepatic steatosis and fibrosis were significantly alleviated in the synbiotic group compared to the placebo group (Mofidi et al., 2017). The two trials suggest that synbiotic supplementation can improve NAFLD symptoms in patients with high, normal, or low BMI.

However, another randomized, double-blind, placebo-controlled phase II trial conducted in 2020 (NCT01680640), in which participants were given synbiotic agents containing fructo-oligosaccharides and Bifidobacterium spp. for 10–14 months, showed that synbiotic supplementation had no effect on liver fat or fibrosis (Scorletti et al., 2020). This may be because only one combination of probiotics and prebiotics was tested in this trial, which did not have a significant effect on NAFLD.

In conclusion, more trials are needed to study the efficacy of different combinations of synbiotics on NAFLD, as well as the effects of race, sex, age, body type, and other factors on the efficacy of synbiotics.

#### 3.1.6 Thyroid hormone receptor *ß* (THRβ) agonists

Thyroid hormone receptors (THRs), belong to the nuclear receptor superfamily, are ligand-dependent transcription factors regulated by endogenous thyroid hormones. THRs consists of two subtypes, THRα, which is a major subtype in the heart and bone, and THRβ, which is a major subtype in the liver (Sinha et al., 2019). Selective THR- *ß* agonists mainly provide metabolic benefits of thyroid hormones to the liver while avoiding unwanted systemic effects caused by excessive thyroid hormones in the heart and bones (Harrison et al., 2019). Selective THR *ß* agonists are currently being developed including resmetirom (MGL-3196), VK2809 (MB 07811), TERN501, ASC41 for the treatment of NAFLD. It has been proved that normal hypothyroidism and subclinical hypothyroidism are independent risk factors for NASH and advanced fibrosis, which is one of the reasons why THR *ß* agonists are still being studied (Kim et al., 2018).

##### 3.1.6.1 Resmetirom (MGL-3196)

In a 36-week randomized, double-blind, placebo-controlled, phase 2 study (NCT02912260), 125 patients with NASH and ≥10% hepatic steatosis confirmed by biopsy were randomly assigned to Resmetirom (80 mg) and placebo group. The results showed that compared with placebo, resmetirom had statistically significant effects in reducing liver enzymes, liver fat, lipoprotein (a), atherogenic lipids, inflammation and fibrosis markers, and improving NASH of liver biopsy. Generally, Resmetirom is well tolerated, the most common adverse events are nausea and diarrhea. This study provides a theoretical basis for three ongoing phase 3 trials (NCT04197479, NCT04951219, NCT03900429) to further evaluate the efficacy and safety of resmetirom in the treatment of NAFLD (Harrison et al., 2019). The results of the clinical trials will guide the pharmacological treatment of NAFLD.

### 3.2 Lipotoxicity and cell death-based targets

Lipotoxic mediators may include free (unesterified) cholesterol, saturated free fatty acids, diacylglycerol, lysophosphatidylcholine, sphingolipids, and ceramide (Farrell et al., 2018). Lipotoxicity can induce endoplasmic reticulum (ER) stress in hepatocytes, which restores ER homeostasis by activating the unfolded protein response (UPR) (Lebeaupin et al., 2018). If ER homeostasis cannot be restored, prolonged activation of the unfolded protein response may initiate apoptotic cell death by upregulating C/EBP homologous protein (CHOP) (Hu H. et al., 2018). Mitochondrial dysfunction and ER stress caused by lipotoxicity lead to an imbalance between oxidants and antioxidants, resulting in oxidative stress (Arroyave-Ospina et al., 2021). ER stress and oxidative stress can lead to the activation of c-Jun N-terminal kinase (JNK) and other pathways, thus resulting in apoptosis. The synergistic effect of ER stress and oxidative stress leads to the occurrence and development of NAFLD (Fujii et al., 2018). The research progress on drugs targeting lipotoxicity and cell death is summarized in Table 2.

**TABLE 2:** Mechanism and Research Progress of drugs targeting lipotoxicity and cell death.

Drug name	Drug targets	NCT number	Current research progress	Status	Reference
Ornithine Aspartic Acid	Targeted oxidative stress. Ammonia-lowering action	NCT05042245	Phase Ⅳ	Recruiting	https://clinicaltrials.gov
Resveratrol	Targeted oxidative stress. Regulate Sirtuin-1 autophagy pathway. Reduce ER stress	NCT02030977	Phase Ⅱ/Ⅲ	Completed	Faghihzadeh et al. (2014)
Vitamin E	Targeted oxidative stress. Improve inflammation and fibrosis	NCT01792115	Phase Ⅱ	Completed	Podszun et al. (2020)
Silymarin	Targeted endoplasmic reticulum stress. Improve inflammation and fibrosis	NCT02006498	Phase Ⅱ	Completed	Wah Kheong et al. (2017)
Niacin	Targeted oxidative stress. Improve hepatic steatosis, inflammation and lipid accumulation	NCT04330326	Phase Ⅱ	Unknown	https://clinicaltrials.gov
Anthocyanins	Targeted oxidative stress. Decrease sembp1c. Induced PPARα Activity	NCT01940263	Phase Ⅰ	Completed	Zhang et al. (2015)
Bicyclol	Inhibition of MAPK and NF-κB signal path	-	Preclinical study	Completed	Zhao et al. (2021)
Piceatannol	Targeted oxidative stress. Reduce fat accumulation	-	Preclinical study	Completed	Yang et al. (2020)
Polydatin	Targeted oxidative stress. Improve inflammation and fibrosis	-	Preclinical study	Completed	Li et al. (2018)

PPAR α, peroxisome proliferator-activated receptor α; MAPK, mitogen-activated protein kinase; NF-κB, nuclear factor-κB.

#### 3.2.1 Vitamin E

Oxidative stress is a promising therapeutic target in NAFLD. Vitamin E is an effective antioxidant that reduces liver inflammation (Nagashimada and Ota, 2019). A mate-analysis of the effect of vitamin E supplementation on adult patients with NAFLD showed that the value of liver enzymes was reduced by vitamin E administration compared to the placebo (−5.71 IU/L, 95% CI: −9.49 to −1.93 for AST and −7.37 IU/L, 95% CI: −10.11 to −4.64 for ALT) (Vadarlis and Antza, 2021). In an experiment on the accumulation pathway of intrahepatic triglyceride (IHTG) in the liver (NCT01792115), 20 patients with NAFLD were randomly sorted into groups to receive 200, 400, or 800 IU/D vitamin E for 24 weeks; 50% of patients had a ≥25% relative decrease in IHTG (Podszun et al., 2020). The antioxidant, anti-inflammatory, and antiapoptotic properties and ease of use of vitamin E make it a practical treatment choice for patients with NAFLD (Perumpail et al., 2018). Vitamin E is a highly effective antioxidant obtained through diet; it can inhibit the development of oxidative stress and inflammation and thus, has a positive impact on NAFLD. However, long-term use of vitamin E may lead to an increase in all-cause mortality, so more accurate methods are still needed to prove its safety (Perumpail et al., 2018).

#### 3.2.2 Silymarin

Silymarin, a bioactive component of milk thistle, has anti-inflammatory, anti-fibrosis, and antioxidant effects (Federico et al., 2017). Silymarin can reduce ER stress proteins GRP78 and XBP-1, thereby relieving the NAFLD symptoms; it can be used to treat diseases caused by ER stress (Sahin and Bagci, 2020). A meta-analysis of silymarin in patients with NAFLD showed that silymarin treatment resulted in a statistically significant reduction in transaminase levels compared to a placebo (Kalopitas et al., 2021). According to the clinical manifestations of NAFLD, a low-calorie diet and physical activity supplemented with silymarin are the correct methods for NAFLD treatment (Colica et al., 2017).

A randomized, double-blind, placebo-controlled trial (NCT02006498)was conducted in adults with biopsy-confirmed NASH and NAFLD activity scores (NAS) ≥ 4. Patients were randomly divided into two groups and administered silymarin (700 mg; n = 49) or a placebo (n = 50) thrice daily for 48 weeks. The results showed that silymarin could improve liver fibrosis compared to the placebo, but the NAS score decreased by no more than 30% in patients with NASH(Wah Kheong et al., 2017). A phase II trial (NCT00680407) was conducted at five medical centers in the United States to test a patented standardized silymarin preparation (Legalon^®^, Rottapharm Madaus, Mylan) on patients with NAS≥ 4. Patients were randomly divided into groups that were administered 420 mg (n = 26) or 700 mg (n = 27)of silymarin or placebo (n = 25) three times a day for 48 weeks. The primary endpoint in NAS was a histological improvement of more than two points. The results showed that after 48–50 weeks, 5/26 (19%) of participants in the 420 mg group, 4/27 (15%) in the 700 mg group, and 3/25 (12%) of placebo participants reached the primary endpoint (*p* = 0.79), indicating that silymarin (Legalon ^®^) had no significant benefit in terms of histological improvement (Navarro et al., 2019). Silymarin alone did not significantly improve NAS; however, it can resist the development of fibrosis and reduce the level of aminotransferase. Therefore, the combination of silymarin is a more feasible method for NAFLD treatment. Therapeutic doses of silymarin have been shown to be safe for humans, but more clinical trials for pregnant women are needed. And it must be cautious when combined with drugs with narrow therapeutic windows (Soleimani et al., 2019). Further clinical trials are needed to better use silymarin.

### 3.3 Inflammation-based targets

Liver inflammation is caused by innate immune cells, mainly macrophages. Monocytes are mainly recruited through CCR2-CCL2 interaction (Baeck et al., 2012). Other chemokine pathways, such as CXCR3-CXCL10, CCR1-CCL5, and CCR8-CCL1, may also contribute to monocyte recruitment (Tacke, 2017). Ly-6C + monocyte infiltration is a key factor in NAFLD, which promotes hepatitis and subsequent fibrosis progression. Liver macrophages of patients with NASH have an obvious inflammatory phenotype, which may be the result of excess lipids and free fatty acids (Jindal et al., 2015). Accumulation of liver macrophages and inflammation are considered hallmarks of the progression of liver disease in patients with NASH. Experimental evidence shows that inflammatory macrophages promote NASH progression through a variety of mechanisms, including liver cell steatosis (Navarro et al., 2015), inflammatory lymphocytes (Wehr et al., 2013), angiogenesis (Ehling et al., 2014), and liver fibrosis (Ju and Tacke, 2016). The research progress on drugs targeting inflammation is summarized in Table 3.

**TABLE 3:** Mechanism and Research Progress of drugs targeting inflammation.

Drug name	Drug targets	NCT number	Current research progress	Status	Reference
Berberine	Inhibit nod-like receptor family Pyrin domain containing 3 inflammasome activation and pyroptosis in nonalcoholic steatohepatitis *via* the ROS/TXNIP Axis	NCT03198572	Phase Ⅳ	Recruiting	https://clinicaltrials.gov
Selonsertib	ASK1 inhibitor. Improve inflammation and fibrosis	NCT03053063	Phase Ⅲ	Terminated	Harrison et al. (2020)
Namodenoson	ADORA3 Agonist. Induce a robust anti-inflammatory effect in the liver through de-regulation of the Wnt/β-catenin pathway	NCT02927314	Phase Ⅱ	Completed	Safadi et al. (2021)
Amlexanox	IKKε and TBK1 inhibitor. Inhibit the activation of KCs and induce polarization of KCs toward the M2 phenotype	NCT01975935	Phase Ⅱ	Completed	https://clinicaltrials.gov
JKB-121	TLR4 antagonist. Decrease fibrosis and inhibit hepatic stellate cell activation and collagen expression *in vitro*	NCT02442687	Phase Ⅱ	Completed	https://clinicaltrials.gov

ASK1, apoptosis signal-regulating kinase 1; A3AR, A3 adenosine receptor; IKKε, IκB kinase epsilon; TBK1, activator-binding kinase 1; TLR4, toll-like receptor 4.

#### 3.3.1 Selonsertib

Selonsertib (formerly GS-4997) is a selective inhibitor of apoptotic signal-regulated kinase 1 (ASK1) activated by oxidative stress. Selonsertib acts on the effector kinases p38 and c-Jun N-terminal kinase *via* the mitogen-activated protein kinase pathway, which regulates liver pro-inflammatory and pro-fibrotic changes (Harrison et al., 2020). In a randomized, open-label, phase II trial, 72 patients were randomly assigned to receive 24 weeks of treatment with six or 18 mg selonsertib alone, six or 18 mg selonsertib with 125 mg simtuzumab, or 125 mg simtuzumab alone. Simtuzumab, a humanized monoclonal antibody directed against lysyl oxidase-like molecule 2, has been proved to be ineffective at reducing hepatic fibrosis (Harrison et al., 2018a). Evaluation of the treatment effectiveness involved liver biopsy, magnetic resonance imaging (MRI), and non-invasive liver injury markers. After 24-week of treatment, selonsertib-treated patients had higher rates of fibrosis improvement and lower rates of fibrosis progression than that in patients treated with simtuzumab alone. The proportion of patients with at least a one-stage reduction in fibrosis after 24 weeks of treatment in the 18 mg selonsertib group was 13 of 30 (43%; 95% CI, 26–63); compared to eight of 27 in the 6 mg selonsertib group (30%; 95% CI, 14–50); and two of 10 in the simtuzumab-alone group (20%; 95% CI, 3–56) (Loomba et al., 2018). Selonsertib improved liver fibrosis in a considerable number of patients with NASH and stage 2 or three fibrosis, indicating its potential as a future treatment for NAFLD. However, in two large, randomized, phase III trials in patients with bridging fibrosis or compensated cirrhosis due to NASH, treatment for 48 weeks with the ASK1 inhibitor selonsertib was ineffective (Harrison et al., 2020). Selonsertib monotherapy had no effect in these trials, but when used in combination with other drugs, there can be a significant effect. Research is still underway into selonsertib combination treatment.

#### 3.3.2 Namodenoson

Namodenoson is a selective A3 adenosine receptor (A3AR) agonist. It is highly selective for A3AR in pathological liver cells and can produce powerful anti-inflammatory effects (Cohen et al., 2011). The A3AR belongs to the family of the Gi-protein associated cell membrane receptors. And the anti-inflammatory mechanism of the A3AR agonist involves deregulation of the NF-κB signaling pathway and induction of apoptosis of inflammatory cells (Ochaion et al., 2008). Namodenoson can also inhibit ischemia-reperfusion injury and act as a protective agent in the liver (Ohana et al., 2016). Namodenoson exhibits a triple mechanism of action in the liver. It exerts anti-inflammatory and anti-fibrosis effects by activating A3AR and relaxing the regulation of the NF-κB and Wnt/β-catenin pathways (Fishman et al., 2019). A phase II double-blind trial randomized 60 patients with NAFLD (ALT≥60IU/L) (1:1:1) and divided them into oral namodenoson groups administered 12.5 mg b.d. (n = 21), 25 mg b.d. (n = 19), or placebo (n = 20) groups for 12 weeks (total follow-up: 16 weeks). The primary efficacy endpoint was the normalization of serum ALT levels. After 12 weeks of treatment, serum ALT levels decreased in a dose-dependent manner with namodenoson over time. At week 12, 31.6% of the namodenoson 25 mg b.d. group and 20.0% of the placebo group achieved ALT normalization (*p* = 0.405). At week 16, 36.8% and 10.0% (*p* = 0.038) patients achieved ALT normalization in the namodenoson 25 mg b.d. and placebo groups, respectively. In addition, approximately one-quarter (23.8%) of the 12.5 mg b.d. group achieved normalization of serum ALT levels at week 16 (Safadi et al., 2021). During the entire phase II clinical trial period, namodenoson significantly reduced liver fat and inflammation and was well tolerated without serious adverse reactions. Various liver parameters, such as ALT and AST levels, improved significantly. Namodenoson also has cardioprotective and neuroprotective characteristics that may compensate NAFLD patients with comorbid cardiovascular and diabetic diseases (Cross et al., 2002). In conclusion, namodenoson can be a potential treatment option for NAFLD.

### 3.4 Extracellular matrix deposition anti-fibrosis-based targets

Hepatic fibrosis is characterized by the overexpression and accumulation of extracellular matrix (ECM) proteins in the liver as a result of the parenchymal cell damage caused by different hepatotoxic agents and mechanisms. The main stroma-producing cell types in liver fibrosis are hepatic stellate cells (HSCs), which are used for lipid storage, and which transform into myofibroblasts (Tacke and Weiskirchen, 2021). However, prolonged liver injury activates them from a resting state to a pro-inflammatory/pro-fibrotic fibroblast-like phenotype, which can contribute to ECM remodeling (Gabbia et al., 2021). The pathways that activate HSCs are diverse, including transforming growth factor (TGF)-β, the hormone fibroblast growth factor 21 (FGF21) (Le et al., 2018) and newly discovered pathways such as hedgehog, autophagy, free cholesterol, YAP1, hepcidin, and nuclear/G-protein-coupled receptor-mediated signals (Tsuchida, 2019).

In response to these mechanisms, drug targets can be divided into two categories: inhibition of fibril formation and enhancement of fibrinolysis. Cenicriviroc is an antagonist of C-C motif chemokine receptor-2/5 (CCR2/5) and is anti-fibrotic as it inhibits collagen activation, HSCs activation, and proliferation (Puengel et al., 2017). However, studies on drugs that increase fibrinolysis are lacking. A recent study shows that the HMGB one peptide can induce fibrinolysis (Nojiri et al., 2021). Drugs targeting extracellular matrix deposition anti-fibrosis and their research progress are summarized in Table 4.

**TABLE 4:** Mechanism and Research Progress of drugs targeting extracellular matrix deposition antifibrosis.

Drug name	Drug targets	NCT number	Current research progress	Statue	Reference
Belapectin	gal-3 inhibitor. Reduce liver fibrosis and portal hypertension	NCT04365868	Phase Ⅲ	Recruiting	https://clinicaltrials.gov
Aramchol	SCD1 inhibitor. Directly inhibit HSCs and induce the protective gene PPARc	NCT04104321	Phase Ⅲ	Recruiting	https://clinicaltrials.gov
Cenicriviroc	CCR2/CCR5 dual inhibitor. Inhibit macrophage accumulation in the liver and ameliorates fibrosis	NCT03028740/NCT02217475	Phase Ⅱ/Ⅲ	Terminated/Completed	Anstee et al. (2020); Ratziu et al. (2020)
Pegbelfermin	PEGylated analogue of FGF21. Improve insulin sensitivity, lipid and adiponectin concentrations, and biomarkers of fibrosis	NCT02413372	Phase Ⅱ	Completed	Sanyal et al. (2019)
NGM282	Non-tumorigenic analogue of FGF19. Retain the ability to suppress CYP7A1 without activating STAT3 signalling	NCT02443116	Phase Ⅱ	Completed	Harrison et al. (2018b)
PXS-5382	LOXL2 inhibitor.Decrease cell numbers, proliferation, colony formations and cell growth, induce cell cycle arrest and increase apoptosis	NCT04183517	Phase Ⅰ	Completed	https://clinicaltrials.gov
Oxy210	hedgehog and TGF- *ß* signalling inhibitor. Attenuated expression of TGF-β-induced pro-fibrotic genes *in vitro*	-	Preclinical study	Completed	Hui et al. (2021)
HMGB1 peptide	Induce anti-inflammatory macrophages and inactivate T cells	-	Preclinical study	Completed	Nojiri et al. (2021)
JT 003	AdipoR1/AdipoR2 dual agonist. improve insulin resistance in high fat diet induced NASH mice and suppress hepatic stellate cells activation in CCl	-	Preclinical study	Completed	Xu et al. (2020)
TM5275	plasminogen activator inhibitor-1 inhibitor. Ameliorat the development of hepatic fibrosis and suppressed the proliferation of activated hepatic stellate cells	-	Preclinical study	Completed	Noguchi et al. (2020)
DZNep	EZH2 Inhibitor. inhibit multiple histone methylation modifications	-	Biological Testing	Completed	Zeybel et al. (2017)

CCR2/CCR5, C-C chemokine receptors type 2 and 5; gal-3, galectin-3; TGF- β, transforming growth factor-beta; AdipoR1/AdipoR2, adiponectin receptors 1/2; PAI-1, plasminogen activator inhibitor-1; HSCs, hepatic stellate cells; LOXL2, Lysyl oxidase-like 2.

#### 3.4.1 Cenicriviroc

C chemokine receptor types 2 (CCR2) and 5 (CCR5) mediate fibrosis by recruiting inflammatory monocytes and macrophages and activating lymphocytes and hepatic stellate cells (Lefebvre et al., 2016; Tacke, 2018). Cenicriviroc (CVC), a dual antagonist of CCR2/CCR5, has anti-inflammatory and anti-fibrosis effects in animal models (Pedrosa et al., 2020). A randomized, double-blind, multinational CENTAUR study randomly assigned 298 participants with NASH to take 150 mg CVC or a placebo daily for 2 years (NCT02217475). Of these 298 participants, 242 entered the second year of the trial, 24% switched to CVC, and 17% remained on the placebo. Twice as many CVC patients whose fibrosis improved in year one maintained the benefit in year two compared with patients receiving the placebo. However, over 2 years, the same proportion of patients taking CVC or placebo met the primary endpoint of hepatic histology improvement in NAS by more than two points with no fibrous deterioration. Interestingly, patients with stage F2 or F3 fibrosis were more likely to benefit from treatment. Moreover, the safety and tolerability of CVC are similar to those of the placebo (Friedman et al., 2018b; Ratziu et al., 2020). Based on these results, the efficacy and safety of CVC will be comprehensively evaluated in a global, multicenter, randomized, double-blind, placebo-controlled study (AURORA, NCT03028740) in patients with stage F2 or F3 NASH (Anstee et al., 2020). However, the highly anticipated Phase III clinical trial of AURORA was terminated in early 2021. This is due to the lack of validity of some of its findings. At present, the combination therapy of CVC and Tropifexor has entered the phase II clinical study and achieved good results (Pedrosa et al., 2020). We do not know whether CVC monotherapy is feasible and need to wait for follow-up studies.

#### 3.4.2 Belapectin

Galectins belong to the non-integrin *ß*-galactoside-binding lectin family. Increased galectin-3 levels are strongly associated with fibrosis, cancer, and inflammation. However, the exact mechanisms of its action are currently unknown (Harrison and Dennis, 2018; Al Attar et al., 2021). Belapectin (GR-MD-02) is a galectin-3 inhibitor derived from plants. It had good anti-fibrotic effects in mouse models and was well-tolerated and safe in phase I clinical trials (Harrison et al., 2016).

The hepatic venous pressure gradient (HVPG) can be used to assess improvements in portal pressure. Portal hypertension is associated with mortality in patients with NASH. In a randomized, double-blind, placebo-controlled phase II trial (NCT02462967), patients with NASH, cirrhosis, or portal hypertension were randomly assigned to two groups. The treatment groups were administered two or 8 mg/kg belapectin (n = 54 each) biweekly and compared to the placebo group (n = 54) for 52 weeks. These results did not meet the primary or secondary endpoints for either dose. This indicates that belapectin was not significantly correlated with HVPG, the incidence of cirrhotic complications, or liver histology. However, a subgroup analysis showed that 2 mg/kg belapectin reduced HVPG and prevented the development of new varices in another group of patients without esophageal varices (Chalasani et al., 2020). The results of phase I and IIb trials indicate that treatment should target patients with NASH cirrhosis without esophageal varices. Moreover, a phase IIb/III study will further investigate the benefits of belapectin in the prevention of other liver-related complications.

#### 3.4.3 3β-arachidinoyl amide cholic acid

3β-arachidinoyl amide cholic acid (Aramchol) is fatty acid-bile acid conjugate that partially inhibits hepatic stearoyl-CoA desaturase (SCD1) protein expression (Bhattacharya et al., 2021). In animal experiments, Aramchol reduced hepatic steatosis and improved steatohepatitis and fibrosis (Iruarrizaga-Lejarreta et al., 2017; Aljohani and Khan, 2019). Aramchol was shown to be safe and well tolerated in an early clinical trial and significantly reduced liver fat in NAFLD patients (NCT01094158) (Safadi et al., 2014).

In a 52-week, double-blind, placebo-controlled, phase 2b clinical trial, 247 NASH patients were randomized to 400 or 600 mg/day Aramchol or placebo (NCT 02279524). There was a significant reduction in liver fat in the 400 mg group (*p* = 0.045) and a reduction in liver triglycerides in the 600 mg group compared with placebo (*p* = 0.066). However, aramchol 600 mg decreased liver triglycerides without meeting the prespecified primary end point for statistical significance. Happily, aramchol ameliorated liver histology in patients with T2D or prediabetes with histologically confirmed steatohepatitis and with high disease activity and precirrhotic stages of fibrosis. (Ratziu et al., 2021). A Phase 3 trial is currently underway, allowing patients to receive a different regimen (Aramchol 300 mg bid) to achieve higher exposures in an attempt to detect the magnitude difference observed in this study (NCT04104321). We will continue to pay attention to the follow-up experimental results of this drug and have certain expectations for it.

## 4 Combination therapy

Owing to the limited efficacy and side effects of monotherapy, other methods are being investigated to treat NAFLD. Moreover, there are various therapeutic targets for NAFLD. An increasing number of studies have shown the limitations of monotherapy and suggested instead developing combination therapies. The advantages of combination therapy include improving the response rate and reducing the side effects of drugs. For example, the response rate of drugs used in NASH monotherapy is <32% compared with a placebo. Combination therapy may increase the response rate by converting non-responders or partial responders into responders. Additionally, combination therapy may treat the side effects of a given drug through the effects of additional drugs or by reducing its dose-dependent side effects without compromising its efficacy, i.e. adding another drug can allow for a reduction in the dose of the original drug (Dufour et al., 2020). Many trials have investigated combination therapies for NAFLD. The drug combinations for NAFLD are listed in Table 5.

**TABLE 5:** Mechanism and research progress of combination therapy for NAFLD.

Drug name	Mechanism	NCT number	Current research progress	Status	Reference
Vitamin E and Pioglitazone	Improve inflammation and steatosis	NCT01002547	Phase Ⅳ	Completed	Bril et al. (2019)
Vitamin E and Hydroxytyrosol	Improve oxidative stress, insulin resistance, and steatosis	NCT02842567	Phase Ⅲ	Completed	Mosca et al. (2021)
Pentoxiphylline and Vitamin E	Reduce hepatic inflammation and fibrosis	NCT01384578	Phase Ⅲ	Withdrawn	Kedarisetty et al. (2021)
Tropifexor and Cenicriviroc	Improve fibrosis and steatohepatitis	NCT03517540	phase Ⅱb	Completed	Pedrosa et al. (2020)
Obeticholic acid and atorvastatin	Ameliorate the OCA-induced side effects on plasma lipoprotein spectrum	NCT02633956	Phase Ⅱ	Completed	Pockros et al. (2019)
Cilofexor and Firsocostat	Improve fibrosis	NCT03449446	Phase Ⅱ	Completed	Loomba et al. (2021)
Omega-3 fatty acids and probiotic	Reduce liver fat, improve metabolism and inflammation	NCT03528707	Clinical phase	Completed	Kobyliak et al. (2018)
N-3 fatty acids and phytosterol esters	Improve the efficacy on hepatic steatosis and decrease the levels of inflammation	ChiCTR1800014419	Clinical phase	Completed	Song et al. (2020)
Synbiotic and sitagliptin	Improve the efficacy on steatosis and inflammation of liver	-	Clinical phase	Completed	Sayari et al. (2018)
Vitamin E and Vitamin D and Silybin	Reduce hepatic inflammation and fibrosis	NCT04640324	Clinical phase	Completed	Federico and Dallio, (2019)
Vitamin E, artichoke leaf extract and metformin	Improve inflammation and fat accumulation	IRCT2017040429278N1	Clinical phase	Completed	Majnooni et al. (2021)
Obeticholic acid and elafbranor	Improve the interaction with lipid treatment and insulin signal transduction, inhibit immune response and reduce the formation of extracellular matrix	-	Preclinical study	Completed	Roth et al. (2019)
SUMOylation inhibitors and FXR agonists	SUMOylation inhibitors rescue FXR signaling and thereby increasing the efficacy of OCA against HSC activation and fibrosis	-	Preclinical study	Completed	Zhou and Cui, (2020)
Metformin and genistein	Reduce blood glucose and the level of TG in the liver and affect the pathway of gluconeogenesis	-	Preclinical study	Completed	Zamani-Garmsiri et al. (2021)
Luteolin and lycopene	Improve the cell viability and lipid accumulation of HepG2 cells and primary hepatocytes induced by PA, and improve weight gain and hepatocyte steatosis	-	Preclinical study	Completed	Zhu et al. (2020)
Glucagon-like peptide-1 receptor agonist and obeticholic acid	Reduce liver steatosis, inflammation and fibrosis	-	Preclinical study	Completed	Jouihan et al. (2017)

Sirt1, Sirtuins1; AMPK, AMP-activated protein kinase; TG, triglyceride.

### 4.1 Combination of OCA and atorvastatin

Several studies have demonstrated the effectiveness of OCA in NAFLD treatment (Kowdley et al., 2018; Younossi et al., 2019b; Kjærgaard et al., 2021; Kulkarni et al., 2021). However, OCA can increase the concentration of serum total cholesterol and low-density lipoprotein cholesterol (LDLc) and decrease the concentration of high-density lipoprotein cholesterol (HDLc), which limits its clinical application (Neuschwander-Tetri et al., 2015). Statins can reduce LDL and triglyceride (TG) levels and slightly increase HDL (McTaggart and Jones, 2008). Thus, a combination of OCA and statins may alleviate the side effects of OCA on the plasma lipoprotein spectrum.

A randomized, double-blind, placebo-controlled phase II study (NCT02633956) was conducted to investigate the efficacy and safety of OCA combined with atorvastatin in NAFLD treatment. In this study, 84 patients with NASH were randomly divided into three treatment groups (5, 10, or 25 mg OCA once daily) and one placebo group for 16 weeks. Each group was additionally administered atorvastatin (10 mg/day) once a day beginning at week 4. In the fourth week, the mean LDLc and mean LDL particle concentration (LDLpc) in all OCA groups were higher than the baseline, and in the eighth week, 10 mg atorvastatin treatment resulted in LDLc and LDLpc levels lower than the baseline in all OCA groups. Additionally, atorvastatin improved the increase in serum total cholesterol and the reduction in HDL caused by OCA(Pockros et al., 2019). Thus, atorvastatin can ameliorate OCA-induced side effects in the plasma lipoprotein spectrum. The combination of OCA and atorvastatin seems to be a good choice for the treatment of NAFLD, trials with larger sample sizes and longer study periods are required to further evaluate the efficacy of combination therapy with OCA and atorvastatin.

### 4.2 Combination of FXR agonists and SUMOylation inhibitors

Hepatic stellate cells (HSCs) play key roles in the pathological development of liver fibrosis (Kowdley et al., 2020). HSCs activation is a marker of liver fibrosis. Loss of lipid droplets (LDS) is a key step in promoting HSC activation (Pawella et al., 2014; Kory et al., 2016; Koyama and Brenner, 2017; Jiang et al., 2021). Plin1 was identified as an FXR target gene responsible for stabilizing LDS in HSCs. Furthermore, FXR agonists can stabilize LDS in HSCs by activating the FXR target gene plin1, but SUMOylation gradually increases during the activation of HSCs, thus inhibiting the FXR signaling pathway. Hence, SUMOylation inhibitors can reduce FXR signaling, which amplifies the effect of FXR agonists, thereby improving the therapeutic efficacy of FXR agonists for activated HSCs and liver fibrosis.

Compared to individual administration, a combination of OCA and spectinomycin (SP, a SUMOylation inhibitor) increased lipid storage and reduced all pre-fibrosis biomarkers. Combined treatment with OCA and SP significantly decreased levels of serum ALT and AST, but there was no significant decrease upon treatment with OCA or SP alone (Čopič et al., 2018). Combined treatment significantly reduced serum aminotransferase levels and improved the histological characteristics of the liver, including steatosis, inflammatory infiltration, ballooning, and a series of liver fibrosis diseases. In addition, the SUMOylation inhibitor ginkgolic acid had a significant effect on activated HSC when combined with OCA. Overall, a combination of a SUMOylation inhibitor and FXR agonist is a promising treatment for liver fibrosis, including NASH, indicating another potential treatment option for utilizing FXR against various types of liver fibrosis.

### 4.3 Combination of vitamin E and silymarin

Vitamin E exhibits antioxidant, anti-inflammatory, and anti-apoptotic activities, along with a favorable clinical profile, making it an appropriate therapeutic choice for NAFLD. However, vitamin E does not affect liver fibrosis. Therefore, vitamin E combined with other anti-fibrotic agents (silymarin) may provide improved treatment for patients with NAFLD.

In a randomized clinical trial of silymarin with vitamin E for NAFLD treatment, 36 patients were randomly divided into two groups. The first group was administered 540.3 mg *Silybum marianum* Gaerth in tablet form and 36 mg vitamin E once daily (Eurosil 85^®^, MEDAS SL) and underwent lifestyle changes, including eating a low-calorie diet. The second group only underwent lifestyle changes. The results show that gamma glutamine transpeptidase levels, fatty liver index, and liver fibrosis scores decreased in both groups. However, the patients in the first group who did not lose 5% of their bodyweight still showed improvement in these parameters, whereas the patients in the second group did not. Therefore, both this drug treatment and lifestyle improvements can improve disease status (Aller et al., 2015), i.e., a combination of silymarin and vitamin E can improve liver function in patients with NAFLD. However, there are few clinical research results, and further clinical trials are needed to determine the efficacy of combination therapy.

## 5 Discussion

NAFLD has no obvious symptoms until it reaches an advanced stage, at which point many patients are diagnosed. Liver biopsy has long been the “gold standard” for diagnosing NAFLD; however, it has well-known limitations, such as sampling bias and serious complications. Therefore, in clinical practice, it is best to adopt an accurate non-invasive method to diagnose different disease stages. Non-invasive tests can also be used as screening tools in the general population to identify high-risk groups. In recent years, non-invasive methods for diagnosing NAFLD have been widely developed, for example, serum biomarkers and imaging techniques. In the current study, US and H-MRI could be used to accurately diagnose NAFLD. US is widely used in clinical practice owing to its low cost and practicality. VCTE was the first FDA-approved elastography model used to assess the severity of fibrosis. It has short processing times and good reproducibility and can be combined with biomarkers to further improve diagnostic performance. More effective non-invasive biomarkers and imaging techniques are needed in the future to diagnose NAFLD and track disease progression.

Overall, studies show that it is not only the selection of a drug that must be considered to maximize the effects of a treatment scheme, other influencing factors determining the final outcome should also be considered. Individualized therapy is the selection of the best drug regimen based on individual patients’ factors. In the case of NAFLD, the main considerations are the patient’s disease state, physiological characteristics, and genetic characteristics at the molecular level.

First, the pathophysiological state was considered, i.e., the screening of appropriate target populations based on disease status. For example, belapectin is more effective in patients with NASH but without esophageal varices, so patients with NASH without esophageal varices can be treated with belapectin. Another example is vitamin E, which is indicated in patients with NASH but without T2DM. In addition to the different disease states, the stage of the disease of the target population must be considered. For example, studies have confirmed that CVC is more beneficial for patients with NASH at stages F2–F3. Moreover, there is heterogeneity in drug therapy for patients with NAFLD of different races, ages, sexes, and body types, and the impact of these factors should be considered when selecting participants.

It is also important to weigh efficacy against adverse effects. For example, some adverse effects of pioglitazone are a major cause of discontinuation, including fluid retention, fractures, and, more seriously, an increased risk of hospitalization for heart failure. Pioglitazone should be administered with caution, particularly in patients with heart disease. Therefore, when individualizing medication, especially in special populations, a thorough assessment of its efficacy and side effects should be considered, including timing, dose, frequency, duration, combination, and the need to change medication. This should be evaluated regularly during treatment, and the continuation or change of the treatment regimen should be decided based on the results.

In addition, the determination of gene polymorphisms can provide relevant information on treatment response. In recent years, multiple genome-wide associations and large candidate gene studies have enriched our knowledge of the genetics of NAFLD. Notably, the I148M PNPLA3 variant has been identified as a major co-genetic determinant of NAFLD (Wang et al., 2011; Valenti and Dongiovanni, 2017). Precision medicine can modulate the activity of a specific gene (PNPLA3) in specific organs in specific patient populations. And other NASH-related genes may provide targets for future intervention strategies. In addition, a genome-wide analysis study identified several loci associated with response to OCA in NASH patients. These variant-associated genetic variants may improve the effectiveness and accuracy of selecting NASH patients for OCA therapy, as these variants can increase NASH resolution (Gawrieh et al., 2019). NASH patients with these genetic variants may then be directed to individualized treatment. However, more research and evidence are needed before speculating whether genotyping can be used to guide treatment decisions in patients at risk for NASH.

Finally, considering the complexity of the pathophysiology of the disease and the heterogeneity of the individual, the detection of specific biological markers should be considered when administering drugs to identify genetic polymorphisms, develop more precise dosing regimens and achieve better therapeutic results (Dufour et al., 2020).

The pathogenesis of NAFLD is complex and involves many factors. Due to the complexity of NAFLD pathophysiology, many targets have potential therapeutic effects, including carbohydrate and lipid metabolism, fibrosis, and inflammation. At present, there have been many studies on NAFLD drugs, most of which are monotherapies, and their efficacy is generally unsatisfactory. Recently, an increasing number of studies have been conducted on NAFLD combination therapy, and some drug combinations have entered clinical phases II or III, indicating their feasibility. Although combination therapy can be more effective than monotherapy and could potentially reduce side effects, its clinical application still has limitations.

The first limitation is the choice of drug. Many drugs are potentially effective against NAFLD, and it is difficult to select a suitable combination from a large number of possibilities. Moreover, the selection of drugs should not be limited to those that are effective against NAFLD; drugs that have no individual efficacy but have synergistic effects with others should not be excluded. Therefore, many studies are needed to screen and combine various drugs, which will be a lengthy process. Second, although combination therapy can be more efficacious, it may also produce further side effects. Therefore, attention should be paid to both side effects and efficacy. In addition, the increase in the types of drugs used in combination therapy also brings some difficulties to the design of the trials, including the allocation of trial groups and the selection of participants. Despite these challenges, combined drug therapy remains a promising treatment option for NAFLD.

It is worth mentioning that dual-target drugs are another current research hotspot. These drugs can act on two or more targets simultaneously and exhibit significant efficacy. Some patients who are difficult to treat with single-target drugs can respond well to these drugs. Therefore, the design and application of dual-target drugs for NAFLD may be beneficial.

## 6 Conclusion

NAFLD pathogenesis is a complicated process that has not yet been fully elucidated. In this review, we briefly introduced diagnostic methods, therapeutic targets, and drugs related to NAFLD. In particular, we focused on the role of carbohydrate and lipid metabolism, lipotoxicity, cell death, inflammation, and fibrosis as potential therapeutic targets for NAFLD. We also summarized the clinical research progress in terms of drug development and combination therapy. Numerous drugs have progressed into clinical studies and have achieved excellent results in clinical NAFLD treatment. However, owing to the complexity of NAFLD and drug side effects, no effective drugs are available on the market. Additionally, combination therapy may have curative effects on NAFLD and NASH by affecting multiple pathways. In the near future, renewed and sustained efforts must be made to provide patients with NAFLD and NASH with safe and effective drugs.
